# Wind-driven spume droplet production and the transport of *Pseudomonas syringae* from aquatic environments

**DOI:** 10.7717/peerj.5663

**Published:** 2018-09-26

**Authors:** Renee B. Pietsch, Hinrich Grothe, Regina Hanlon, Craig W. Powers, Sunghwan Jung, Shane D. Ross, David G. Schmale III

**Affiliations:** 1 Biological Sciences, Virginia Polytechnic Institute and State University (Virginia Tech), Blacksburg, VA, United States of America; 2 School of Plant and Environmental Sciences, Virginia Polytechnic Institute and State University (Virginia Tech), Blacksburg, VA, United States of America; 3 Institute of Materials Chemistry (E165), TU Wien, Vienna, Austria; 4 Civil and Environmental Engineering, Virginia Polytechnic Institute and State University (Virginia Tech), Blacksburg, VA, United States of America; 5 Biomedical Engineering and Mechanics, Virginia Polytechnic Institute and State University (Virginia Tech), Blacksburg, VA, United States of America

**Keywords:** Wind, Aerosol, Ice nucleation, Droplet, Bacteria, *Pseudomonas syringae*, Aquatic microbiology, Bioprecipitation, Air-water interface, Bubble bursting

## Abstract

Natural aquatic environments such as oceans, lakes, and rivers are home to a tremendous diversity of microorganisms. Some may cross the air-water interface within droplets and become airborne, with the potential to impact the Earth’s radiation budget, precipitation processes, and spread of disease. Larger droplets are likely to return to the water or adjacent land, but smaller droplets may be suspended in the atmosphere for transport over long distances. Here, we report on a series of controlled laboratory experiments to quantify wind-driven droplet production from a freshwater source for low wind speeds. The rate of droplet production increased quadratically with wind speed above a critical value (10-m equivalent 5.7 m/s) where droplet production initiated. Droplet diameter and ejection speeds were fit by a gamma distribution. The droplet mass flux and momentum flux increased with wind speed. Two mechanisms of droplet production, bubble bursting and fragmentation, yielded different distributions for diameter, speed, and angle. At a wind speed of about 3.5 m/s, aqueous suspensions of the ice-nucleating bacterium *Pseudomonas syringae* were collected at rates of 283 cells m^−2^ s^−1^ at 5 cm above the water surface, and at 14 cells m^−2^ s^−1^ at 10 cm above the water surface. At a wind speed of about 4.0 m/s, aqueous suspensions of *P. syringae* were collected at rates of 509 cells m^−2^ s^−1^ at 5 cm above the water surface, and at 81 cells m^−2^ s^−1^ at 10 cm above the water surface. The potential for microbial flux into the atmosphere from aquatic environments was calculated using known concentrations of bacteria in natural freshwater systems. Up to 3.1 × 10^4^ cells m^−2^ s^−1^ of water surface were estimated to leave the water in potentially suspended droplets (diameters <100 µm). Understanding the sources and mechanisms for bacteria to aerosolize from freshwater aquatic sources may aid in designing management strategies for pathogenic bacteria, and could shed light on how bacteria are involved in mesoscale atmospheric processes.

## Introduction

Terrestrial environments are estimated to release between 40 and 1,900 Gg bacteria per year (Burrows et al., 2009). Microorganisms also aerosolize from aquatic surfaces in freshwater and saltwater aquatic environments, but little is known about the abiotic and biotic processes that govern aerosolization from these environments (Blanchard, Syzdek & Weber, 1981; Gantt & Meskhidze, 2013; Lewis & Schwartz, 2004; Veron, 2015). Water surfaces produce droplets that contain microorganisms, liberating microorganisms (Baylor & Baylor, 1980) into the atmosphere where they may be involved in atmospheric processes, including cloud formation as cloud condensation nuclei (Dinger, Howell & Wojciechowski, 1970; Park et al., 2014) or ice nuclei (Baldy & Bourguel, 1987; Baylor et al., 1977; Bigg & Leck, 2008; Blanchard & Woodcock, 1957; Blanchard, 1989; Morris et al., 2014). Aerosolized microorganisms can also affect the earth’s radiation budget (Gabric et al., 2005; Haywood, Ramaswamy & Soden, 1999; Park et al., 2014) and disease spread (Polymenakou, 2012). Once airborne, microbes can be carried through the atmosphere over great distances (Schmale III & Ross, 2015). Marine algae and diatoms have been reported 160 km downwind from the sea coast in rime frost on top of Mt. Washington in New Hampshire (Baylor & Baylor, 1980). Approximately 10% of microbes in the boundary layer at a given time were still airborne four days later giving them the potential to travel up to 11,000 km before deposition (Mayol et al., 2014).

Microorganisms are released from aquatic environments inside small droplets, which are described as fluid volumes bound by immiscible interfaces characterized by an interfacial tension (Fernando, Bourouiba & Bush, 2012). Droplets are produced by several mechanisms including bubble bursting and spume droplets tearing off of breaking waves by fragmentation (Wu, 1981). In bubble bursting air bubbles rise and burst at the sea surface producing film and jet droplets which enter the atmosphere (Baldy & Bourguel, 1987; Blanchard, 1989; O’Dowd & De Leeuw, 2007; Sellegri et al., 2006; Wu, 1981). Bubbles form predominantly from breaking waves as air is entrained into the surface of the water column producing bubbles (Bigg & Leck, 2008; Blanchard, 1989; Blanchard & Woodcock, 1957). As these bubbles rise regions of foam form at the surface called whitecaps. Strong winds increase wave-breaking (whitecaps) and fragmentation bubble production (Blanchard & Woodcock, 1957). Near-surface wind speed increases with height, and is commonly reported at the reference height of 10 m above the water surface (Mueller & Veron, 2009a; Ovadnevaite et al., 2014). Breaking waves form at wind speeds of *U*_10_ = 3–4 m/s (Blanchard, 1989; Hanson & Phillips, 1999; Monahan & Muircheartaigh, 1986). Whitecap coverage increases rapidly with more than approximately the third power of wind speed (Blanchard, 1989). Several factors influence the relationship between whitecap coverage and wind speed including wind history (Callaghan et al., 2008). Bubble bursting is considered the primary means of microbial aerosolization from aquatic surfaces (Blanchard, 1989). Fragmentation droplets only form at higher wind speeds, while bubble bursting droplets are smaller and occur at high and low wind speeds (Andreas, 2002; Monahan et al., 1983; Wu, 1981). Due to a combination of smaller vertical angle and larger droplet size, fragmentation droplets tend to fall back into the water quickly limiting their potential for aerosolization (Wu, 1981).

The size distribution of bubbles in the water and at the water surface has been characterized (Cipriano & Blanchard, 1981; Spiel, 1998), as well as the relationship between bubble size and the number, size, ejection speed and ejection height of droplets (Blanchard, 1989; Cipriano & Blanchard, 1981; Spiel, 1995). Size distributions for droplets have been studied as well (Spiel, 1994). Estimates of production flux based on fragmentation droplets (spume droplets) have been proposed (Mueller & Veron, 2009b).

A variety of experimental methods and modeling approaches have been used to study droplet production. These methods include holography and photography techniques (Koga, 1982; Leifer, De Leeuw & Cohen, 2000; Resch, Darrozes & Afeti, 1986), field experiments (Wu, Murray & Lai, 1984), and laboratory experiments with a wind wave tank (DeMott et al., 2016; Koga, 1981; Veron et al., 2012). Droplet production has been modeled from a simulation tank (Monahan, Davidson & Spiel, 1982) and a numerical model has been developed (Andreas, 1998). Several of the experimental studies have focused on artificial methods of generating bubbles, such as entraining air to produce bubbles (Cipriano & Blanchard, 1981; Wu, 1989), plunging waterfalls (Stokes et al., 2013), and use of a continuous plunging jet (Salter et al., 2014). Bubble bursting has been studied with porous glass or metal frits (Mårtensson et al., 2003). Research to examine fragmentation droplets, and the relative contributions from the two mechanisms together, is lacking. A model for fragmentation droplet generation was compared to a model based on field experiment observations and another based on wind wave tank observations (Wu, 1993). The three models showed considerable variability and wide discrepancies indicating fragmentation droplets are not well understood and further characterization is necessary.

Bubble population at the air-water interface controls the flux of aerosol into the atmosphere (Baldy & Bourguel, 1987; Salter et al., 2014). The surface microlayer is enriched with microorganism effecting aerosolization and increasing the amount of bacteria that can aerosolize (Cunliffe et al., 2012). Bacterial aerosol emissions from sea water have been studied in a laboratory simulation looking at the quantity, type, and size of bacteria (Fahlgren et al., 2015; Hultin et al. 2010). Global aerosols from the ocean have been estimated between 1,300–3,300 Tg/yr (Andreae, 1995; De Leeuw et al., 2011). The atmospheric boundary layer contains an estimated 6 × 10^4^ to 1.6 × 10^7^ microbes m^−2^ of ocean (Mayol et al., 2014), but not all of these microbes necessarily aerosolized from the ocean; they may have aerosolized from other sources. Better estimates of the aerosol production rate—specifically how wind speed influences the aerosolization rate—would be helpful in estimating the impact of aerosolized microorganisms. The global average 10-m wind speed over the ocean is 6.6 m/s; that over the continents (including inland aquatic sources) is 3.3 m/s (Archer & Jacobson, 2005). Microorganisms range widely in size; bacterial cells are typically 0.3 to 10 µm (Cole & Cook, 1998). Small bacteria dominate in surface waters, such as the bacterium *Pseudomonas syringae,* which has dimensions of 1–5 µm × 0.5–1.5 µm (Monier & Lindow, 2003). Microorganisms cross the air-water interface in droplets, thus studying droplet production can give a better understanding of microbial aerosolization from aquatic environments. Bubble bursting droplets and fragmentation droplets have mostly been studied separately.

**Figure 1:**
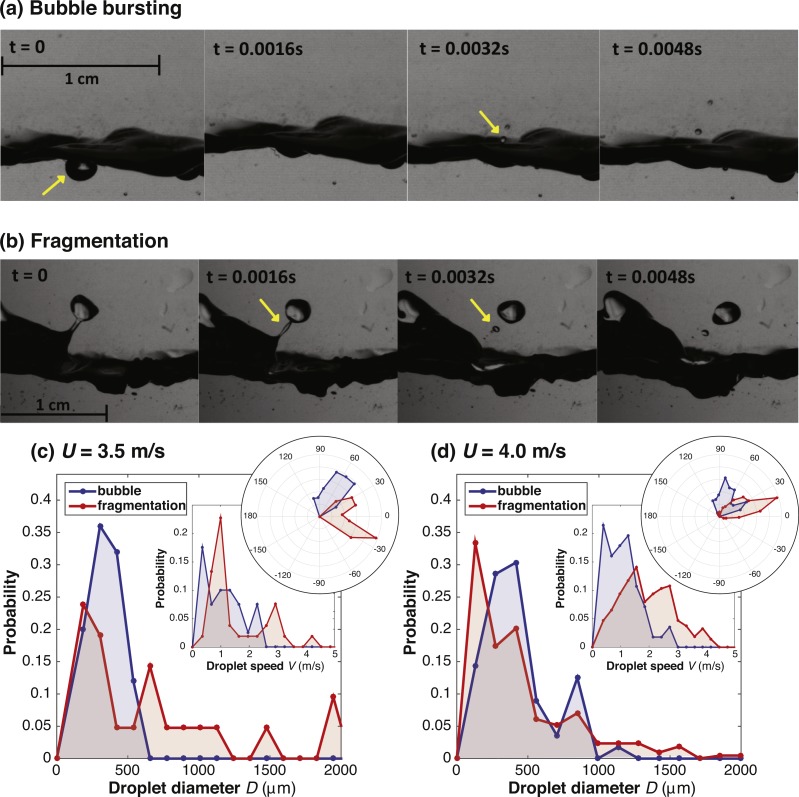
High speed images recorded at 6,250 fps. Series of high speed images recorded at 6,250 fps (i.e., 0.00016 s between frames) showing (A) bubble bursting droplet formation and (B) fragmentation droplet formation. The wind direction is to the right. Each series shows four images 10 frames apart. The sub-surface bubble in the first frame of (A) produces droplets seen in the third frame. The ligament identified in the second frame of (B) breaks up into droplets. See the video version at the following URL: https://youtu.be/lGZmx4h0yMA. (C–D) Probability distributions for droplet diameter (*D*), droplet ejection speed (*V*), and ejection angle at wind speeds *U* = 3.5 and 4.0 m/s (measured at 2.5 cm above water surface) for bubble bursting droplets (blue) and fragmentation droplets (red). The downwind direction is zero degrees and the radial increments are 0.05 on a relative scale. For 3.5 m/s, bubble and fragmentation droplets have population sizes *n* = 26 and *n* = 33, respectively. For 4.0 m/s, the population numbers are *n* = 57 and *n* = 221, respectively.

Here, we simulated the action of the wind on an aquatic system, and characterized the droplets produced, in terms of diameter, ejection velocity and angle, as a function of both wind speed and production mechanism (bubble bursting or fragmentation; see Fig. 1). Experiments were conducted with aqueous suspensions of *P. syringae* to determine the number of cells transported at different heights (5 and 10 cm) under different wind speeds (3.5 and 4 m/s). We considered a range of 10-m wind speeds consistent with the global mean wind speeds (Archer & Jacobson, 2005), including the lowest wind speeds above the critical value necessary for wave-induced aerosolization of droplets. We hypothesized that the characteristics of the ensemble of droplets produced change with wind speed, specifically, the droplet production number flux and mass flux are zero below a critical wind speed and beyond the critical value they increase with wind speed. Moreover, we hypothesized that the certain characteristics of the droplet ensemble may be well fit by an analytical distribution. Droplet characteristics can then be used to calculate the production mass flux and aerosolization potential for bacteria, such as *P. syringae,* from aquatic environments. Desai et al. (2009) reported an increasing trend of stronger surface winds (based on comparisons of land and lake (buoy) measurements) across the largest freshwater lake in the world (Lake Superior, about 0.22 m/s increase in surface wind speed per decade since 1985). New information is needed on the sources and mechanisms for bacteria to aerosolize from freshwater aquatic sources, particularly in the context of climate change and extreme weather. Such efforts could shed light on how bacteria originating from aquatic sources may be involved in mesoscale atmospheric processes.

**Figure 2:**
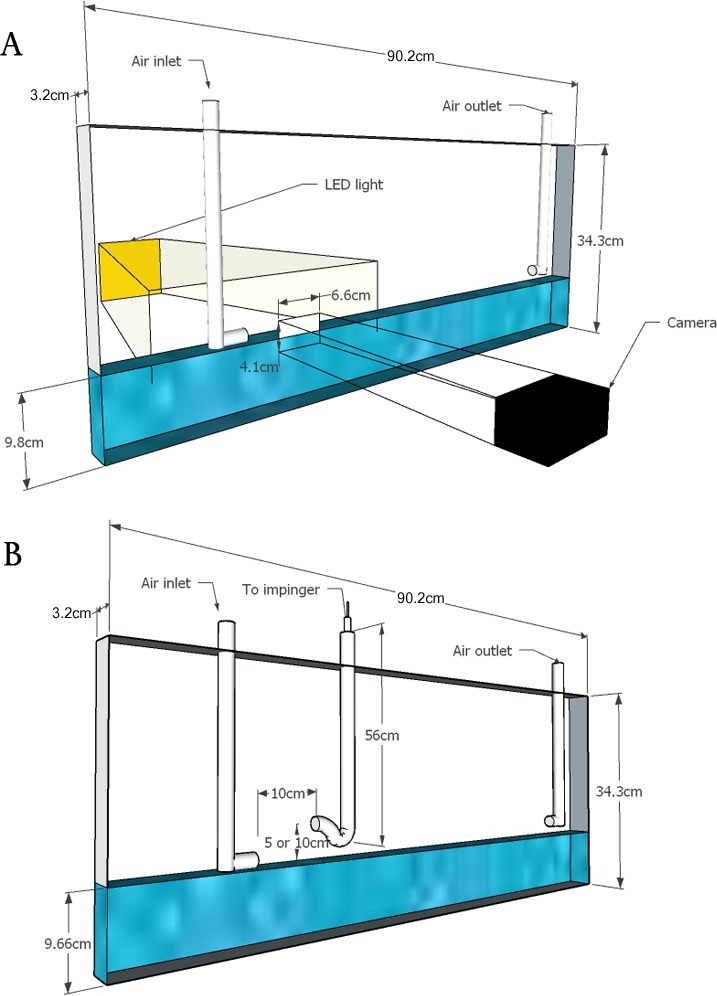
Schematic of flumes used in laboratory experiments. Schematic showing the experimental design of Flume A for imaging experiments (A) and Flume B for experiments to monitor the transport of *P. syringae* (B). For Flume A, the camera viewed an area of 6.6 cm by 4.1 cm at the air-water interface. For both flumes, the air outlet was connected to a vacuum which pulled air across the water surface and the air inlet allowed air to enter the system.

## Methods

### Flume design for imaging experiments (Flume A)

The flume for imaging experiments (Flume A) was constructed from 0.635 cm width Plexiglas with inner dimensions of 90.2 cm ×3.2 cm ×34.3 cm, as shown in Fig. 2A. The flume was a closed system with one 1.27 cm diameter inlet pipe reaching to the surface of the water and one 1.27 cm diameter outlet pipe exiting near the water surface. The inlet pipe had a nozzle shape designed to direct water away from the side of the flume minimizing droplets landing on the flume side. The flume was filled with deionized water to a height of 9.8 cm. Experiments were conducted at ambient room temperature. The flume outlet was connected to a vacuum that pulled air across the water surface simulating wind moving on the water surface. The vacuum was controlled with a 5 Amp FS-5F single pole 120 V rotary dimmer switch (Lutron, Coopersburg, PA, USA). A 29 series II multimeter (Everett, WA, USA) with a resolution of 0.1 V and accuracy of ±1% was used to measure the voltage of the dimmer switch. A EA-3010 anemometer (LaCrosse Technology, La Crosse, WI, USA) with a resolution of 0.1 m/s was used to measure wind speed. The wind speed *U* was measured at *z* = 2.5 cm above the water surface and on the downwind edge of the camera’s field of view. Measured wind speeds were nearly identical (and thus stable) up to a distance of 40 cm downwind from the inlet nozzle (well beyond the viewing window of the camera) for flume flow rates of 3.0 m/s. At distances greater than 40 cm from the inlet nozzle, the windspeed increased up to a factor of three at the outlet (located 60 cm downwind from the inlet). Consequently, the flume demonstrated spatially uniform (or non-accelerating) flow of near constant velocity near the water surface, within the viewing window of where the wind-driven spume droplets were observed by the camera. The outlet valve to the vacuum was adjusted until the anemometer fluctuated by ±0.2 m/s of the target speed. After an initial calibration was made, the wind speed was controlled only with the multimeter. A SpectroLED-14 light (Genray, New York, NY, USA) was placed behind the flume. A Photron FASTCAM Mini UX100 camera (Photron, San Diego, CA, USA) with a micro-Nikkor 105 mm f/28 lens (Nikon, New York, NY, USA) was used to record video data at 6250 frames per second (0.16 ms between frames) in 0.9 s increments with a resolution of 1,280 × 800 pixels. The camera field of view was 6.6 cm of length by 4.1 cm of height. The resolution was 52 µm per pixel. The depth of field allowed the entire 3.8 cm width of the flume to be in focus. Videos were captured at four wind speeds (*U* = 3.5 m/s, 4.0 m/s, 4.5 m/s and 5.0 m/s). At 3.5 m/s, 12 video increments were recorded on 2 days (*n* = 122). At 4.0 m/s, 12 video increments were recorded on 4 days (*n* = 563). At 4.5, m/s four video increments were recorded on 2 days (*n* = 672). At 5.0, m/s 3 video increments were recorded on 2 days (*n* = 771). Assuming a power-law for the wind profile (Etkin, 1981), we have *U*_10_∕*U* = (*z*_10_∕*z*)^*α*^ where *U* is the wind speed measured at height *z* (in m) above the water surface, and *U*_10_ is the wind speed at the reference height *z*_10_ = 10 m. While there is significant uncertainty in the exponent *α*, which depends on atmospheric stability, surface roughness and nearby obstacles, we use a value of 0.1 which is appropriate for near-neutral conditions over a large body of water (Hsu, 1988). We did not account for any potential wall effects of our narrow flume (Williams, 1970).

### Flume design for transport experiments for aqueous suspensions of *P. syringae* (Flume B)

The flume initially designed for the imaging experiments was modified (Flume B) to allow for a series of transport experiments with aqueous suspensions of *P. syringae*. Inner dimensions of Flume B were 90.2 cm × 3.2 cm × 34.3 cm. A droplet-sampling trap (v-shaped with a horizontal inlet) was used to capture cells of *P. syringae* from an aqueous suspension in the flume. The orifice of the trap had a diameter of 1.27 cm, and the inlet was 4 cm long, measured from the center of the trap (Fig. 2B). The trap was designed to be 56 cm tall, to prevent droplets from reaching the top end due to precipitation and deposition at the walls. When the trap was installed for experiments with *P. syringae*, additional turbulence was introduced in the system. Therefore, all wind speed measurements for the *P. syringae* experiments were conducted without the trap installed. At the top of the trap tube, a flexible hose of 0.64 cm diameter connected the tube to an impinger filled with 20 ml of 0.2 µm filter sterilized water. A vacuum was used to pull air through the impinger, and was controlled by a valve on the vacuum line to achieve a steady flow rate. The trap system was surface sterilized with 70% ethanol prior to each experiment. Two different trap extension lengths allowed sampling at two different heights, 5 and 10 cm above the suspension of *P. syringae* in the flume. All parts of the trap system were rinsed with 1 ml wash of sterile distilled water to test for any bacteria deposited on the different pieces of the trap.

### Image analysis with Flume A

Video images were converted to black and white. The centroid and the centroid trajectory of each droplet were found across a stack of images. Diameters of the droplets were determined from the pixel area of the centroid data using the mean of the first ten images to minimize error due to small variations. Variation in the estimated diameter across images was typically ±5%, increasing to ±15% for the smallest resolved diameters (52 µm). Ejection speed was determined from the droplet centroid displacement between images, as near as possible to the point of ejection from the water surface. Variation in the estimated speed, determined by the standard deviation in speed estimates across ten images, was ±5%. The angle of the droplets was determined from a line tangent to the droplet trajectory as near as possible to the point of ejection from the water surface, to reduce error due to droplet movement being affected by the wind. Variation in the estimated angle, based on limits of resolution, was ±2 degrees. Droplets were categorized according to the mechanism of droplet production according to visual inspection of the videos, as in Fig. 1: bubble bursting, fragmentation, or unknown. Droplets were classified as ‘unknown’ if the moment the droplet first appears was not caught in the field of view. At *U* = 3.5 and 4.0 m/s, the populations of droplets from the bubble bursting and fragmentation mechanisms were large enough (*n* > 20 in each group) for the two populations to be compared.

### Gamma distribution fit

Gamma distribution for diameters *D*, is given by the probability distribution function,

\begin{eqnarray*}p(D)= \frac{{ \left( \frac{D}{{\theta }_{D}} \right) }^{{k}_{D}-1}{e}^{- \left( \frac{D}{{\theta }_{D}} \right) }}{{\theta }_{D}\Gamma ({k}_{D})} \end{eqnarray*}

where the shape variable is ${k}_{D}={ \left\langle D \right\rangle }^{2}/{\sigma }_{D}^{2}$, the scale variable is ${\theta }_{D}={\sigma }_{D}^{2}/ \left\langle D \right\rangle $, and $\Gamma \left( \cdot \right) $ is the gamma function. This gamma distribution has mean $ \left\langle D \right\rangle $ and variance ${\sigma }_{D}^{2}$. As a pre-processing step to remove significant outliers, we binned the diameters and considered only diameters up to a cutoff diameter, the minimum diameter bin with a number of elements less than 0.02*n*_max_, where *n*_max_ is the number of elements in the bin with the maximum number of elements. The same procedure was followed for the gamma distribution of velocity, yielding the shape variable ${k}_{V}={ \left\langle V \right\rangle }^{2}/{\sigma }_{V}^{2}$ and the scale variable ${\theta }_{V}={\sigma }_{V}^{2}/\langle V\rangle $.

### Experiments with *P. syringae* in Flume B

A 3L suspension of *P. syringae* (ice+ BAV strain #892 (Pietsch et al., 2015)) was diluted from a 4 mL liquid culture grown overnight (12–14 h) at 22 °C in R2 broth (3.15 g/L R2 broth, TEKnova #R0005, Hollister, CA 95023) (Reasoner & Geldreich, 1985). This starting culture was first diluted to an optical density of 0.2 at 600 nm. Control R2A plates were spread with 0.020 mL of a 1 × 10^−6^ dilution of the starter culture to confirm CFU in a countable range of 20–100 colonies per plate. For each flume experiment, a starter culture (optical density of 0.2 at 600 nm, corresponding to a range of 1,000 to 5,000 CFU per mL) was diluted in 10 mM MgSO_4_ to make 3 L of an aqueous suspension of *P. syringae* which was added to the flume. This aqueous suspension (flume solution) was used to fill the flume to a height of 9.66 cm, corresponding to a water volume *V*_Psyringae_ = 2.7 L. Growth of *P. syringae* was determined by CFU counts on two types of agar plate, R2A (R2 broth made with 15% agar (Fisher 9002-18-0)) and KBC (Kings B medium with 15 g/L proteose peptone, 1.5 g/L anhydrous K_2_HPO_4_, 10 mL/L 100% glycerol, 6 mM MgSO_4_) with 24 mM H_3_BO_3_, cephalexin (10 mg/L), and cycloheximide (50 mg/L) (Mohan & Schaad, 1987)). Samples were plated in triplicate and colonies were counted and reported as mean CFU per mL plated. Experiments were conducted with aqueous suspensions of *P. syringae* to determine the number of cells being transported at different heights (5 and 10 cm) under different wind speeds (3.5 and 4 m/s).

Experimental parameters for height and wind speed were held constant for each experiment with a trap collection time of 30 min (1,800 s). Trap collections were sequentially carried out in triplicate. For each 30-minute trap collection, 20 mL of sterile water was loaded into the 50 mL impinger apparatus. Prior to trap collections, 0.10 mL of the flume solution was plated in triplicate on R2A and KBC to obtain tank start values for *P. syringae*. Tank start values were determined for each experiment. Following trap collections, the same plating scheme was carried out for Aerosol Experiments 1, 2, and 4. The end tank aliquot for Aerosol Experiment 3 was not collected. For each 30-minute trap collection, volumes were recovered and 0.10 mL plated in triplicate on R2A and KBC plates. For collected trap volumes less than 2.0 mL, an aliquot of 0.50 mL sterile water was used to rinse the trap. This volume change was calculated as a dilution when determining CFU/mL values for the trap collections in Aerosol Experiment 2 and 4. For each experiment, the three impinger collections were combined and concentrated by filtration through a 0.2 µm filter, followed by an immediate resuspension in 5 mL of sterile filtrate via a 10-minute filter spin in a sterile 100 mL bottle. The combined impinged resuspension was plated in 0.20 mL aliquots in triplicate on R2A and KBC plates.

## Results

### Rate of droplet production

**Figure 3:**
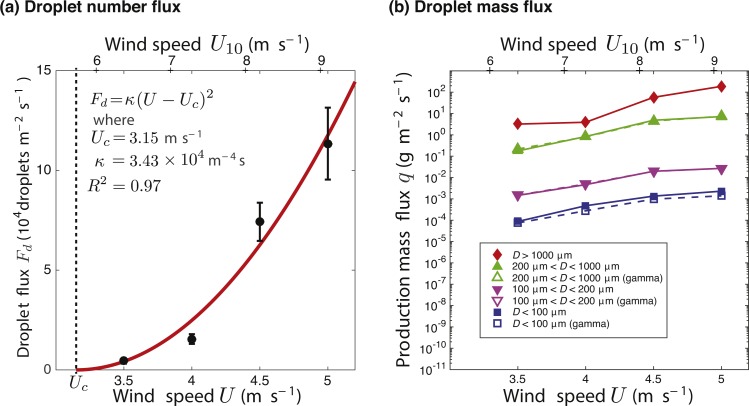
Droplet production rate fit with a second order polynomial. (A) Droplet production rate, *F*_*d*_, in number of droplets m^−2^ s^−1^ of water surface, based on high-speed video at 6250 fps in a wind wave tank at four wind speeds (*U* = 3.5, 4.0, 4.5, and 5.0 m/s) measured at 2.5 cm above the water surface (bottom axis) and calculated to equivalent speeds, *V*_10_, at 10 m above the water surface (top axis). The data is fit with a second order polynomial. Error bars were calculated as standard error of droplet production across the video clips at each wind speed. (B) Production mass flux, *q*, dependence on wind speed based on experimental data (filled symbols and solid lines) and from the gamma distribution fits for diameter (open symbols and dashed lines) for five grouping of the droplets based on droplet diameter *D* (>1,000 µm, 200–1,000 µm, 100–200 µm, and <100 µm). For the >1,000 µm droplets, the gamma distribution does not hold. For the 200–1,000 µm and 100–200 µm groupings, the experimental and gamma distribution data points are nearly on top of each other in this figure.

Rate of droplet production, *F*_*d*_, in m^−2^ s^−1^ of water surface was related to wind speed, *U* in m s^−1^, measured at *z* = 2.5 cm above the water and related to equivalent wind speeds, *U*_10_, at 10 m above the water surface (Fig. 3A and Table 1). The data fit a second order polynomial, ${F}_{d}=\kappa { \left( U-{U}_{c} \right) }^{2}$, for constant parameters *κ* in s m^−4^ and *U*_*c*_ determined from a non-linear curve fit, with an *R*^2^ = 0.97 (it should be noted, however, that this is based on only four windspeeds tested in our study). A critical threshold wind speed of *U*_*c*_ = 3.15 m/s at 2.5 cm height (or equivalently *U*_10*c*_ = 5.7 m/s at 10 m height) was determined, below which there is no droplet production.

**Table 1:** Drops per time and area. The number of drops total and the seconds of video recorded along with the average number of droplets m^−2^ s^−1^ of water surface at each of the four wind speeds.

Wind speed *U* (m/s)	Wind speed at 10 m, *U*_10_ (m/s)	Number of droplets	Total video time (s)	10^4^ droplets m^−2^ s^−1^
3.5	6.4	122	10.8	0.45
4.0	7.3	563	14.4	1.55
4.5	8.2	672	3.6	7.42
5.0	9.1	771	2.7	11.4

**Table 2:** Parameters for droplet diameter and ejection. Parameters for the distribution of droplet diameter *D* and ejection speeds *V* at each of the four wind speeds (*U* at 2.5 cm height and *U*_10_, the equivalent at 10 m), including average $ \left\langle D \right\rangle $, variance ${\sigma }_{D}^{2}$, average $ \left\langle V \right\rangle $, variance ${\sigma }_{V}^{2}$, and the shape *k*, and scale *θ* parameters for the gamma distribution for each, as well as the *R*^2^ of the gamma distribution fit.

*U*(m/s)	*U*_10_ (m/s)	$ \left\langle D \right\rangle $ (µm)	${\sigma }_{D}^{2}$ (µm)^2^	*k*_*D*_	*θ*_*D*_	${R}_{D}^{2}$	$ \left\langle V \right\rangle $ (m/s)	${\sigma }_{V}^{2}$ (m/s)^2^	*k*_*V*_	*θ*_*V*_	${R}_{V}^{2}$
3.5	6.4	387	74,900	2.00	194	0.97	1.33	0.544	3.28	0.407	0.94
4.0	7.3	406	94,100	1.76	231	0.94	1.63	0.781	3.41	0.479	0.94
4.5	8.2	471	121,000	1.85	256	0.96	1.62	0.723	3.64	0.446	0.94
5.0	9.1	525	158,000	1.74	302	0.97	1.55	0.601	4.02	0.387	0.96

### Distributions of droplet diameter, speed, and angle

**Figure 4:**
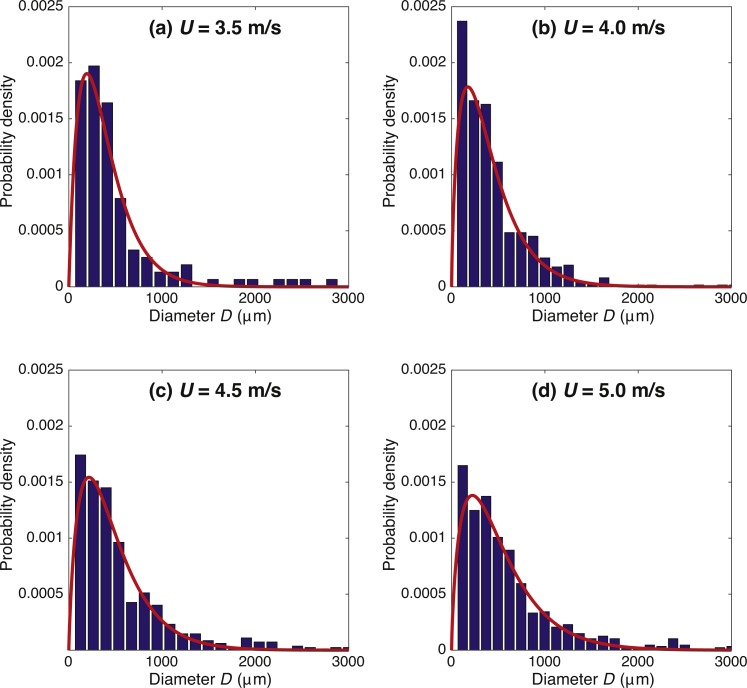
Probability distributions of droplet diameter at each of the four wind speeds ((A–D) *U* = 3.5, 4.0, 4.5, and 5.0 m/s) with a gamma distribution fit (curve), with parameters as given in Table 2.

**Figure 5:**
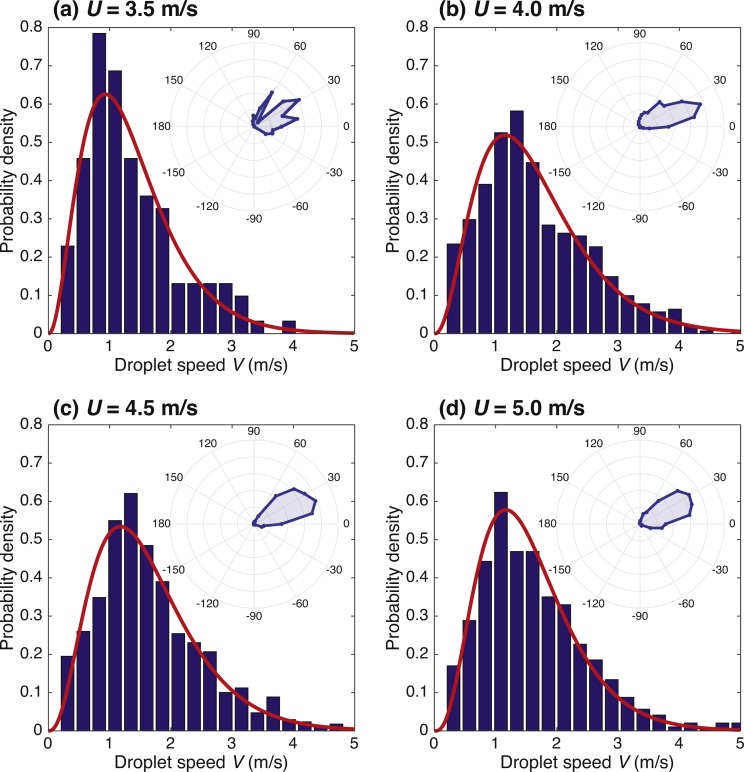
Probability distributions of droplet ejection speed *V* at each of the four wind speeds ((A–D) *U* = 3.5, 4.0, 4.5, and 5.0 m/s) with a gamma distribution fit (curve), with parameters as given in Table 2. The inset for each wind speed is a polar plot of probability distribution for droplet ejection angle. The radial increments are 0.05 on a relative scale. The downwind direction is zero degrees.

Average and variance of droplet diameter, *D*, increased with wind speed; more small and large droplets are produced as well as the largest drop size (*D*_max_ ≈ 5.0 mm for *U* = 3.5 m/s and *D*_max_ ≈ 8.8 mm for *U* = 5.0 m/s). Probability distributions were best fit with a gamma distribution (Fig. 4 and Table 2), calculated from the average $ \left\langle D \right\rangle $ and variance ${\sigma }_{D}^{2}= \left\langle { \left( D- \left\langle D \right\rangle \right) }^{2} \right\rangle $ which provide the shape parameter, *k*_*D*_, and scale parameter, *θ*_*D*_. A log normal distribution (not shown) was also fit to the data (Kolmogorov, 1962; Oboukhov, 1962; Novikov & Dommermuth, 1997), but the gamma distribution was a better representation of the experimental data, giving lower error (having a higher *R*^2^) (Beck & Cohen, 2003; Villermaux, Marmottant & Duplat, 2004). Average droplet diameter and variance generally increased with wind speed. Probability distributions for droplet ejection speed, *V*, at each wind speed also showed a good fit to a gamma distribution (Fig. 5 and Table 2). Probability distributions for the droplet angle for the four wind speeds are given in polar plot form in Fig. 5, where zero degrees is the downwind direction. We see a wider distribution in angle for *U* = 3.5 and 4.0 m/s compared to *U* = 4.5 and 5.0 m/s.

### Distributions of diameter, speed, and angle for bubble bursting and fragmentation droplets

Bubble bursting droplets were observed for 21% (26/122), 10% (285/563), 0.3% (2/672), and 0% (0 out of 771) of the droplets observed at *U* = 3.5, 4, 4.5 and 5 m/s, respectively.  Droplet data at the lowest wind speeds, 3.5 and 4.0 m/s were presented in categories of bubble bursting or fragmentation based on the observed mechanism of droplet production (Figs. 1A–1B). Diameter and speed of bubble bursting and fragmentation droplets indicated, in general, that fragmentation droplets had a wider distribution while the bubble bursting droplets had a narrower distribution, centered around smaller diameters (Figs. 1C–1D). Polar plots for the angle distributions of bubble bursting and fragmentation droplets indicated the bubble bursting droplets in general were between 20° and 100°, while the fragmentation droplets were between −40° and 70°. We were not able to determine the mechanism of production for a majority of the droplets at higher wind speeds, due in part to the noise of droplet splatter on the flume surface. We observed fewer bubble bursting droplets at higher wind speeds, but this may be due to the inability to distinguish bubbles in our experimental set up at higher speeds.

### Droplet production mass flux

Mass flux ranged from 8.7  × 10^−5^ gm^−2^s^−1^ for droplets with diameters *D* < 100 µm at *U* = 3.5 m/s to 200 gm^−1^s^−2^ for droplets with *D* > 1,000 µm at *U* = 5.0 m/s. Mass flux of droplets in each size range (*D* < 100 µm, 100 µm <*D*< 200 µm, 200 µm <*D*<1,000 µm) increased two orders of magnitude with wind speed from 3.5 to 5.0 m/s (Fig. 3B). Mass flux calculated from the gamma distribution showed a good fit with the mass calculated from the experimental data, over the range of diameters where comparisons could be made, indicating that the gamma distribution may be used to extrapolate the mass flux of droplets smaller than the experiment could resolve (*D* < 52 µm).

**Figure 6:**
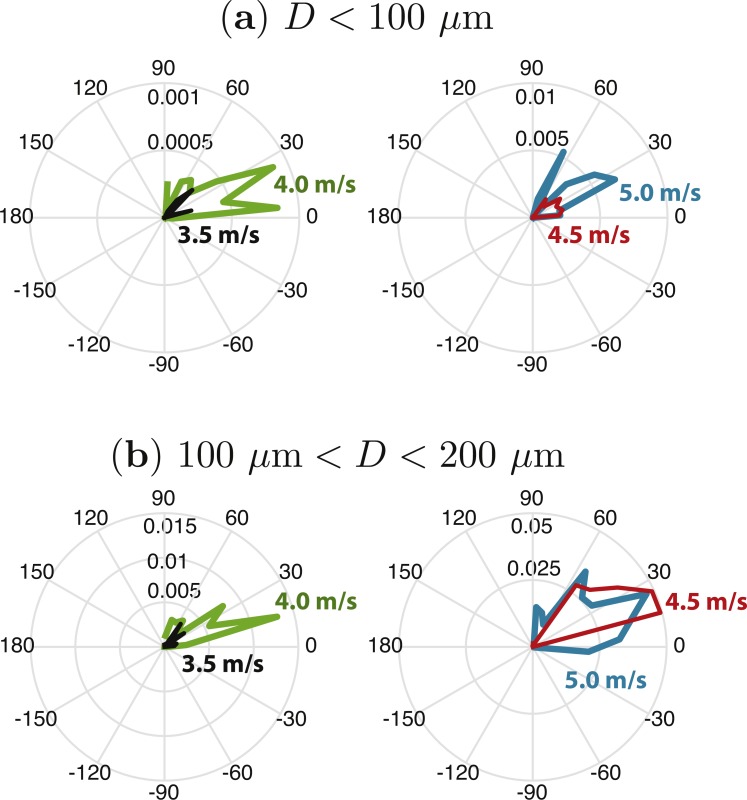
Momentum flux as a function of angle for droplets with diameters (A) *D* < 100 µm and (B) 100 µm <*D*< 200 µm, at four wind speeds (*U* = 3.5, 4.0, 4.5, and 5.0 m/s at 2.5 cm height). The radial increments are measured in g m^−1^ s^−2^ rad^−1^. The total momentum flux for droplets with diameters *D* < 100 µm is 1.4 × 10^−4^, 6.8 × 10^−4^, 2.0 × 10^−3^, 4.8 × 10^−3^ g m^−1^ s^−2^ for the four wind speeds, respectively. The total momentum flux for droplets with diameters 100 µm < *D* < 200 µm is 1.7 × 10^−3^, 7.3 × 10^−3^, 3.5 × 10^−2^, 4.6 × 10^−2^ for the four wind speeds, respectively.

### Droplet production momentum flux

Given their potential role in atmosphere-water momentum exchange (Veron et al., 2012), we report the momentum flux for small droplets (those with the potential to be suspended) as a function of both wind speed and angle (Fig. 6). Values of total momentum flux ranged from 1.4  × 10^−4^ gm^−1^s^−2^ for droplets with diameters *D* < 100 µm at *U* = 3.5 m/s to 4.6  × 10^−2^ gm^−1^s^−2^ for droplets with diameters 100 µm <*D* < 200 µm at *U* = 5.0 m/s.

### Observed and estimated transport of *P. syringae* at different heights under different wind speeds

The start (beginning of each experiment, prior to flume operation) and the end (end of experiment, after flume was turned off) concentrations are reported in Table 3. At a wind speed of about 3.5 m/s, aqueous suspensions of *P. syringae* were collected at rates of 283 cells m^−2^ s^−1^ at 5 cm above the water surface, and at 14 cells m^−2^ s^−1^ at 10 cm above the water surface (Table 3). At a wind speed of about 4.0 m/s, aqueous suspensions of *P. syringae* were collected at rates of 509 cells m^−2^ s^−1^ at 5 cm above the water surface, and at 81 cells m^−2^ s^−1^ at 10 cm above the water surface (Table 3). These trends correspond well to the respective volumes trapped at different wind speeds and at different heights. Increasing the height of the trap (from 5 cm to 10 cm), decreased the trapped volume by a factor of about 11. This is independent from the wind speed. Increasing the wind speed from 3.5 to 4.0 m/s increased the trapped volume by a factor of 3–3.2, which is independent of the height. *P. syringae* were not found above the trap in the flexible hose.

**Table 3:** Observed and estimated flux of cells of *P. syringae* at different heights and wind speeds. Observed and estimated transport of cells of *P. syringae* at two different wind speeds (3.5 and 4.0 m/s) at two different heights (5 and 10 cm). The surface area between the inlet and the mouth of the trap was 0.00318 m^2^; this value was used to compute units of observed flux (cells m^−2^ s^−1^). Tank start refers to the concentration of bacteria at the start of the experiment, and tank end refers to the concentration of bacteria measured at the end of the experiment.

**Exp. #**	**Wind speed(m/s)**	**Height**	**Location**	**Trap or impinger volume (mL)**	**Mean CFU/mLR2A**	**Mean CFU/mL KBC**	**Observed flux, R2A (cells/m2/s)**	**Observed flux, KBC (cells/m2/s)**	**Est. surface flux, R2A cells/m2/s**	**Est. surface flux, KBC (cells/m2/s)**
Exp1	3.5	5 cm	Tank start	1.00	207 ± 8.82	297 ± 60.09			700	1,004
Exp1	3.5	5 cm	Tank end	1.00	493 ± 67.41	437 ± 51.57			1,666	1,477
Exp1	3.5	5 cm	Trap 1	3.20	363 ± 28.48	443 ± 31.80	203	248		
Exp1	3.5	5 cm	Trap 2	3.50	317 ± 13.33	313 ± 92.62	194	192		
Exp1	3.5	5 cm	Trap 3	6.20	417 ± 38.44	417 ± 13.33	451	451		
**Exp1 Mean**				**4.3 ± 0.95**	**366 ± 28.90**	**391 ± 34.72**	**283**	**297**		
Exp2	3.5	10 cm	Tank start	1.00	387 ± 23.33	360 ± 15.28			1,308	1,217
Exp2	3.5	10 cm	Tank end	1.00	697 ± 61.73	613 ± 64.38			2356	2,072
Exp2	3.5	10 cm	Trap 4	0.52	253 ± 20.04	199 ± 15.58	23	18		
Exp2	3.5	10 cm	Trap 5	0.32	125 ± 7.2	123 ± 27.38	7	7		
Exp2	3.5	10 cm	Trap 6	0.35	199 ± 6.33	155 ± 39.49	12	10		
**Exp2 Mean**	** **	** **	** **	**0.4 ± 0.06**	**192 ± 37.10**	**159 ± 22.03**	**14**	**11**		
Exp3	4.0	5 cm	Tank start	1.00	300 ± 11.55	287 ± 8.82			1,422	1,360
Exp3	4.0	5 cm	Tank end	1.00	ND	ND			ND	ND
Exp3	4.0	5 cm	Trap 7	10.50	193 ± 12.02	200 ± 15.28	355	367		
Exp3	4.0	5 cm	Trap 8	15.00	217 ± 12.02	177 ± 17.64	568	463		
Exp3	4.0	5 cm	Trap 9	16.00	217 ± 43.33	140 ± 36.06	606	391		
**Exp3 Mean**	** **	** **	** **	**13.83 ± 1.69**	**209 ± 8.00**	**172 ± 17.48**	**509**	**407**		
Exp4	4.0	10 cm	Tank start	1.00	373 ± 17.64	397 ± 34.8			1,768	1,882
Exp4	4.0	10 cm	Tank end	1.00	750 ± 63.51	537 ± 63.33			3,555	2,545
Exp4	4.0	10 cm	Trap 10	0.58	138 ± 10.89	116 ± 6.45	14	12		
Exp4	4.0	10 cm	Trap 11	1.34	323 ± 35.01	294 ± 27.38	76	69		
Exp4	4.0	10 cm	Trap 12	1.75	506 ± 4.49	363 ± 18.7	155	111		
**Exp4 Mean**	** **	** **	** **	**1.22 ± 0.34**	**322 ± 106.23**	**258 ± 73.58**	**81**	**64**		

We estimated the flux of cells emerging out of the tank water surface by multiplying the total mass production flux by the measured tank cell density. At the 3.5 m/s wind speed, the total mass production flux is 3.4 gm^−2^ s^−1^ and at 4.0 m/s, it is 4.7 gm^−2^ s^−1^. Droplets with diameters exceeding 1000 µm dominate mass production flux at both wind speeds. Based on these mass production flux values and the measured cell density in the tank at the start and end, we can estimate the cell surface flux (Table 3). During the duration of the experiment, the cell density in the tank typically increased by a factor of 2. At the 3.5 m/s wind speed, the estimated surface flux is higher than the measured 5 cm flux by a factor of 2.5–6.1, and higher than the 10 cm flux by a factor of 93–170. At the 4.0 m/s wind speed, the estimated surface flux is higher than the measured 5 cm flux by a factor of 1.5–3.3, and higher than the 10 cm flux by a factor of 26–52.

## Discussion

Though microorganisms are ubiquitous in aquatic environments, little is known about how they get out of the water and into the air. This study examined the characteristics of droplets aerosolized from water at four different wind speeds. Looking at the three parameters together, as the wind speed increases, the diameter range increases, the angle range decreases, and the droplet speed is relatively unchanged. Rate of droplet production scales as $\propto { \left( U-{U}_{c} \right) }^{2}$. In addition, contributions to droplet production from two mechanisms—bubble bursting and fragmentation—were examined at the two lowest wind speeds. These compensating factors all combine to influence the droplet-mediated transport of microorganisms in the atmosphere.

A strong association between droplet production and wind speed was observed, which is consistent with previous literature (Blanchard & Woodcock, 1957; Mueller & Veron, 2009b; Ortiz-Sunslow et al., 2016; Ovadnevaite et al., 2014). Droplet production increased quadratically with wind speed above a critical threshold speed *U*_*c*_. This relationship implies that even small increases in wind speed have the potential to significantly increase the droplet production and mass emission. The critical threshold observed, equivalent to *U*_10*c*_ =5.7 m/s at 10 m height) below which there is no droplet production is in agreement with a previous result (Hamilton & Lenton, 1998).

Droplet diameters were well represented by a gamma distribution at all four wind speeds. Our choice of probability distribution functions is motivated by the field of turbulence. Previously, Kolmogrov, Laudau, and other researchers developed the idea of flow structures in turbulent flows cascading from large to small scales. Below a certain size, structures are dissipated away. The log-normal distribution was proposed by Kolmogorov (1962) and Oboukhov (1962), and the Gamma distribution was proposed by Beck & Cohen (2003). Similarly, in the area of shear-induced fragmentation into droplets, log-normal (Novikov & Dommermuth, 1997) and Gamma (Villermaux, Marmottant & Duplat, 2004) distributions have been proposed. Droplet diameters have previously been studied (Koga, 1981; Resch, Darrozes & Afeti, 1986; Spiel, 1998) as well as the distribution of bubbles before bursting (Leifer, De Leeuw & Cohen, 2000). Droplet size distributions fit to Poisson, gamma, lognormal, exponential curves, and gamma distributions have matched the data best in previous studies (Villermaux, 2007). Gamma distributions have previously been seen for droplet diameters in fragmentation; droplets breaking off of ligaments of water as well as for natural spray (Bremond & Villermaux, 2006; Ling et al., 2017; Villermaux, Marmottant & Duplat, 2004).

The distribution of droplet speed also showed a gamma distribution (Fig. 5), which to our knowledge has not been considered previously in the literature. The 3.5 m/s data had the lowest mean droplet speed with the other three wind speeds showing similar values, indicating that while wind speed has a substantial effect on other parameters, the effect on droplet speed is negligible.

At higher wind speeds the distribution of droplet angles was narrower. For the lower two wind speeds (3.5 and 4.0 m/s) the droplets ranged from below the horizontal (−30°) to slightly beyond the vertical (100°), but at the higher two wind speeds the distribution was more narrow ranging between 0° and 60°. The three parameters (diameter, speed, and angle) were compared in every combination of pairs and no correlation was seen between any of the parameters.

At the two lower wind speeds (*U* = 3.5 and 4.0 m/s) differences were seen in the angle, diameter, and speed distributions of bubble bursting droplets and fragmentation droplets, which could affect aerosolization. A narrower range of both droplet diameters and speeds was observed for bubble bursting data. The mechanism of formation for bubble bursting droplets produces droplets within certain parameters while fragmentation droplets have no upper limit in size (Andreas, 1998). Bubble bursting droplets tend to be ejected closer to the vertical while the fragmentation droplets are torn off of breaking waves with an angle directed near the horizontal, downwind direction.

Large droplets, identified as those above a critical diameter *D*_*c*_ (*D* > *D*_*c*_), are likely to fall quickly back into the water (or neighboring land, such as a shore). Small droplets (*D* < *D*_*c*_) are likely to remain suspended in the atmosphere for long periods of time, since their low settling speeds will be balanced by turbulent updrafts (Cole & Cook, 1998). We note that droplet aerosols with diameters on the order of 100 µm have been shown to remain suspended in air for prolonged periods of time, provided their settling speeds are small relative to turbulent updrafts in indoor conditions (Cole & Cook, 1998; Fernstrom & Goldblatt, 2013). Moreover, the conditions (e.g., wind speeds and gradients) necessary for droplet production in aquatic environments are also conditions for significant air turbulence, including vertical turbulence. Based on a spectral turbulence model (Etkin, 1981), the vertical turbulent velocity is related to the wind at 6 m height via *σ*_*w*_ ≈ 0.1*U*_6_. For the wind speeds considered in this study, interpolated to 6 m, we find *σ*_*w*_ ≈ 0.7 m/s, which balances the terminal velocity of a water droplet of diameter ≈ 200 µm, based on experimental results in still air (Gunn & Kinzer, 1949). We therefore consider *D*_*c*_ ≈ 200 µm, which is consistent with previous reports of the maximum suspended drop size for this range of wind speeds (Mueller & Veron, 2009b).

**Figure 7:**
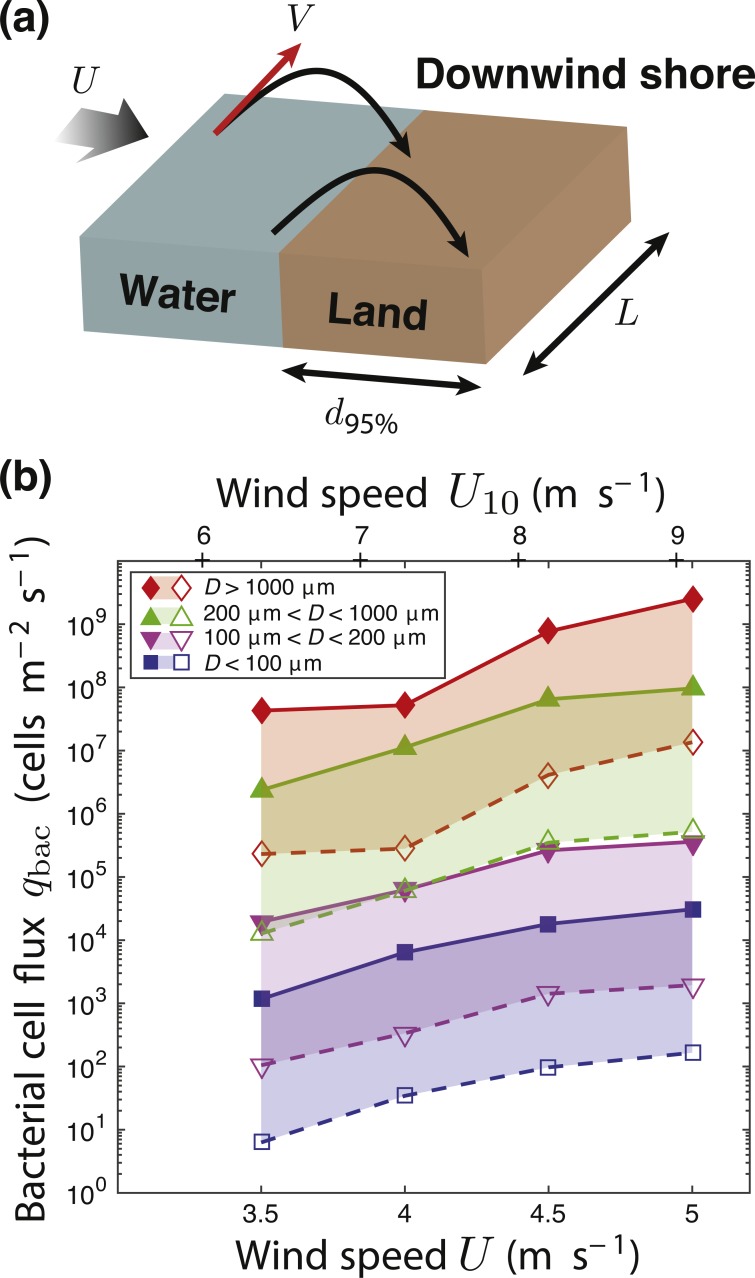
Splash zone. (A) The width of the ‘splash zone’, that portion of the downwind shore adjacent to the body of water that will receive locally released droplets, can be estimated as *d*_95%_, the distance from the shoreline where 95% of the total mass transferred from the water to the land is reached. (B) The potential for bacteria to move across the air-water interface, given as a bacterial cell flux, *q*_*bac*_, based on the droplet mass flux at four wind speeds (reported as *U* at 2.5 cm and *U*10 at 10 m) and at four different droplet size ranges, using the reported bacterial concentrations from literature for freshwater lakes (Bird & Kalff, 1984; Coveney, 1982; Field et al., 1980). For each diameter range, the filled symbols correspond to the high estimate of 1.3 ×10^7^ cells/mL and the open symbols correspond to the lower estimate of 7.2 ×10^4^ cells/mL, with the region between shaded.

When droplet production occurs near land, large droplets (*D* > 200 µm) may spread to adjacent terrestrial surfaces such as shoreline. Angle, diameter, and speed of the droplet, combined with atmospheric conditions, determine the probability of a droplet reaching a given height or horizontal distance. Using a dynamical model along with the measured droplet initial conditions, we can estimate the width of the ‘splash zone’, the downwind area adjacent to the body of water that will receive locally released droplets (Fig. 7A), as well as the mass transfer rate to the splash zone. The drag force on droplets is assumed to have the form,

\begin{eqnarray*}{\mathbi{F}}_{\mathrm{drag}}=- \frac{1}{2} {C}_{D}\rho S \left\vert \mathbi{V }-\mathbi{U} \right\vert \left( \mathbi{V }-\mathbi{U} \right) \end{eqnarray*}

where *ρ* is the density of air, $S= \frac{\pi }{4} {D}^{2}$ is the cross-sectional area of a droplet, ***V*** is the droplet velocity in a local wind of velocity ***U*** which follows the power-law profile assumed before, and for the drag coefficient *C*_*D*_, we use the form adopted by (Flagan & Seinfeld, 1988) appropriate for spherical aerosols,

\begin{eqnarray*}{C}_{D}= \frac{24}{Re} \left( 1+0.15~R{e}^{0.687} \right) , 2\lt Re\lt 500 \end{eqnarray*}

which is the appropriate range of Reynolds number, $Re= \frac{VD}{\nu } $, for the droplet dynamics, where *ν* is the kinematic viscosity of air.

The splash zone width can be estimated as *d*_95%_, the distance from the shoreline where 95% of the total mass transferred from the water to the land is reached. The mass transfer rate per unit length of shoreline will be based on the 95 percentile mass, and varies with the wind speed. For the lowest wind speed above critical, 3.5 m/s, we calculate the splash zone as *d*_95%_ = 0.428 m and a mass transfer rate of 0.828 g s^−1^ m^−1^ of shoreline. The splash zone increases to *d*_95%_ = 1.10 m with a mass transfer rate of 139 g s^−1^ m^−1^ of shoreline at 5.0 m/s.

In a natural system, droplets may contain microorganisms, which can become aerosolized as they cross the air-water interface (Morris et al., 2014). Our flume experiments showed a strong correlation between the mass and volume flux of the droplets and the number flux of aerosolized *P. syringae*. When doubling the height of the trap, the volume collected in the trap decreased by more than an order of magnitude; while a 14% increase in wind speed resulted in tripling the volume collected. The same trend is true for the number of bacteria. At a wind speed of about 3.5 m/s, aqueous suspensions of *P. syringae* were aerosolized at rates of 283 cells m^−2^ s^−1^ at 5 cm above the water surface, and at 14 cells m^−2^ s^−1^ at 10 cm above the water surface. At a wind speed of about 4.0 m/s, aqueous suspensions of *P. syringae* were aerosolized at rates of 509 cells m^−2^ s^−1^ at 5 cm above the water surface, and at 81 cells m^−2^ s^−1^ at 10 cm above the water surface. There is likely a threshold in droplet size below which no bacteria are transported anymore, but this was not quantified in our study.

An estimated concentration of bacteria with the potential of being aerosolized can be calculated from the droplet flux observed in these experiments. We found that bacteria fluxes estimated from the production mass flux and mean bacteria density in the tank were always higher than our measured values (Table 3). In part, this could be due to deposition, as larger droplets fall back into the fluid, since the estimated flux was at the surface while the lowest measured surface flux was at a height of 5 cm above the surface.

The concentration of bacteria in a lake may vary from one lake to another and in different conditions within one lake (Pietsch, Vinatzer & Schmale III, 2017). The total bacterial concentration in lakes between 0 and 2 m deep ranges between 7.2  × 10^4^ and 1.3  × 10^7^ cells/mL (Bird & Kalff, 1984; Coveney, 1982; Field et al., 1980). No known studies have examined the detailed distribution of taxa across different depths, but concentrations of ice-nucleating *Pseudomonas* spp. have been reported for the top 0.2 m of a freshwater lake between 0.11 and 16.24 cells/mL (Pietsch, Vinatzer & Schmale III, 2017). Given the range of total bacteria concentrations, and the droplet mass flux *q* we observed, an estimate of the flux of bacteria moving across the air-water interface, *q*_bac_, can be calculated. Figure 7B shows a low and high estimate based on the range of bacterial concentrations cited above for five size divisions of droplet size at each of the four wind speeds. The droplets of diameter *D* <100 µm are most likely to remain suspended for extended periods and thus most important to examine in the context of capacity to aerosolize bacteria. At *U* = 3.5 m/s, 1.2  × 10^3^ cells leave the water m^−2^ s^−1^ of water surface; this flux increases to 3.1  × 10^4^ cells m^−2^s^−1^for *U* = 5.0 m/s, at the upper estimate for bacterial concentration (the lower bacterial concentration estimate gives 6.3 cells m^−2^s^−1^ and 170 cells m^−2^s^−1^ at the two wind speeds, respectively). If we consider slightly larger droplets with diameters 100 µm < *D* < 200 µm which also have the potential to remain suspended, the range goes from 1.9  ×10^4^ cells m^−2^s^−1^ at *U* = 3.5 m/s to 3.6  × 10^5^ cells m^−2^s^−1^ for *U* = 5.0 m/s based on the upper bacterial concentration estimate (and 100 cells m^−2^s^−1^ to 1. 9 × 10^3^ cells m^−2^s^−1^ using the lower bacterial concentration estimate). Wind speeds are not usually sustained over the whole surface of a body of water, but are intermittent in nature and spatially non-uniform. Given these estimated rates of bacterial flux, the potential exists to aerosolize significant quantities of bacteria, particularly as wind speed increases. Estimates of global aerosolization taken from terrestrial environment measurements in as a flux of bacterial cells m^−2^ s^−1^ of area are 44 to 206 (Burrows et al., 2009). With >70% of the Earth’s surface covered in water, aerosolization from aquatic environments has the potential to be a greater contributor to aerosols than from terrestrial environments. Aller et al. (2005) observed a 15–25-fold enrichment in bacteria during transport from subsurface waters to the surface marine layer. Further work needs to be done to track the amount of bacteria present in aerosolized droplets, as it may not be the same as the concentration in the bulk of the water and is likely higher; concentrations of microorganisms in droplets could exceed the bulk water concentration by 10 to 100 times (Baylor et al., 1977; Blanchard & Syzdek, 1982).

## Conclusions

A series of controlled laboratory experiments were conducted to quantify wind-driven droplet production from a freshwater source for low wind speeds. The rate of droplet production increased quadratically with wind speed. Droplet diameter and ejection speeds fit a gamma distribution. Droplet mass flux and momentum flux increased with wind speed. Two mechanisms of droplet production, bubble bursting and fragmentation, yielded different distributions for diameter, speed, and angle. At a wind speed of about 3.5 m/s, aqueous suspensions of the ice-nucleating bacterium *P. syringae* were collected at rates of 283 cells m^−2^ s^−1^ at 5 cm above the water surface, and at 14 cells m^−2^ s^−1^ at 10 cm above the water surface. At a wind speed of about 4.0 m/s, aqueous suspensions of *P. syringae* were collected at rates of 509 cells m^−2^ s^−1^ at 5 cm above the water surface, and at 81 cells m^−2^ s^−1^ at 10 cm above the water surface. Up to 3.1 × 10^4^ microbial cells m^−2^ s^−1^ of water surface were estimated to leave the water in potentially suspended droplets.

An increased understanding of droplet production may inform the movement of a variety of particles across the air-water interface, and the fate of the particles once they have crossed the interface. In particular, bacteria can move from the water into the air in droplets. With the ubiquitous presence and great diversity of microorganisms in the world, with up to 1 trillion species of bacteria (Locey & Lennon, 2015), we are just beginning to understand all the roles microorganisms play in Earth processes. Some strains of the bacterium *P. syringae* express an ice nucleation protein allowing them to raise the freezing temperature of water. As aerosols in the atmosphere, ice nucleating *P. syringae* may be involved in precipitation processes (Sands et al., 1982). Understanding the sources and mechanisms for bacteria to aerosolize may aid in designing management strategies for pathogenic bacteria, and shed light on how bacteria could be involved in mesoscale atmospheric processes.

##  Supplemental Information

10.7717/peerj.5663/supp-1 Data S1 Raw data for droplets and bacteria counts from lab experiments These are data from lab experiments using the flume and highspeed video.

## Additional Information and Declarations

### Data Availability

The following information was supplied regarding data availability: The raw data are provided in a Data S1.
